# *Momordica charantia* Extract Induces Apoptosis in Human Cancer Cells through Caspase- and Mitochondria-Dependent Pathways

**DOI:** 10.1155/2012/261971

**Published:** 2012-10-04

**Authors:** Chia-Jung Li, Shih-Fang Tsang, Chun-Hao Tsai, Hsin-Yi Tsai, Jong-Ho Chyuan, Hsue-Yin Hsu

**Affiliations:** ^1^Institute of Medical Sciences, Tzu Chi University, Hualien 970, Taiwan; ^2^Department of Anatomy, Tzu Chi University, Hualien 970, Taiwan; ^3^Department of Life Sciences, Tzu Chi University, Hualien 970, Taiwan; ^4^Section of Crop Improvement, Hualien District Agricultural Research and Extension Station, Council of Agriculture, Executive Yuan, Hualien 973, Taiwan

## Abstract

Plants are an invaluable source of potential new anti-cancer drugs. *Momordica charantia* is one of these plants with both edible and medical value and reported to exhibit anticancer activity. To explore the potential effectiveness of *Momordica charantia*, methanol extract of *Momordica charantia* (MCME) was used to evaluate the cytotoxic activity on four human cancer cell lines, Hone-1 nasopharyngeal carcinoma cells, AGS gastric adenocarcinoma cells, HCT-116 colorectal carcinoma cells, and CL1-0 lung adenocarcinoma cells, in this study. MCME showed cytotoxic activity towards all cancer cells tested, with the approximate IC_50_ ranging from 0.25 to 0.35 mg/mL at 24 h. MCME induced cell death was found to be time-dependent in these cells. Apoptosis was demonstrated by DAPI staining and DNA fragmentation analysis using agarose gel electrophoresis. MCME activated caspase-3 and enhanced the cleavage of downstream DFF45 and PARP, subsequently leading to DNA fragmentation and nuclear condensation. The apoptogenic protein, Bax, was increased, whereas Bcl-2 was decreased after treating for 24 h in all cancer cells, indicating the involvement of mitochondrial pathway in MCME-induced cell death. These findings indicate that MCME has cytotoxic effects on human cancer cells and exhibits promising anti-cancer activity by triggering apoptosis through the regulation of caspases and mitochondria.

## 1. Introduction

Cancer is one of the leading causes of death worldwide, accounting for millions of death each year. Previous studies have examined the association between the intake of antioxidant-rich foods and beneficial effects related to the prevention of cancer, cardiovascular diseases, diabetes, and other oxidative-stress-related chronic diseases [1, 2]. The highly reactive and bioactive phytochemical antioxidants in plants are postulated to be responsible, in part, for the protective effects of plant foods. Biochemically active phytochemicals found in plant-based foods also have many powerful biological properties that are not necessarily related to their antioxidant properties [3, 4]. Some cancer patients use agents derived from different plants or nutrients as complementary or alternative medicines, exclusively or concurrently with traditional chemotherapy and/or radiotherapy [5]. Although there are increasing numbers of drugs available for patients with cancer, the effects of many drug treatments are temporary and noncurative. Due to the need for new therapeutic options for cancer therapy, the discovery of food plants with medicinal effects has prompted studies evaluating possible anticancer agents in fruits, vegetables, herbs, and spices [6].

*Momordica charantia *L. (bitter gourd), a member of the family *Cucurbitaceae*, is widely grown in tropical areas and used as a traditional medicine plant indigenous to China. In addition to culinary usage, *M. charantia* is also used in folklore medicine worldwide [6, 7]. *M. charantia* was found to possess antiviral, antibacterial, and immunomodulatory properties and used as a topical remedy for expelling intestinal gas and treating skin problems such as scabies, eczema, and itchy rashes [8–10]. Most often, crude extracts of the bitter gourd fruits were used as hypoglycemic or antidiabetic agents in pathophysiological conditions [11]. In Taiwan, both cultivars and wild-grown *M. charantia* are found. Wild populations with smaller fruit sizes, used as a folklore medicine for a long history by aboriginal people, are native to Taiwan and currently exhibit a sympatric distribution or introgression of cultivars for agricultural purposes [12].* M. charantia* contains an array of components that possess different biological activities. Extract of the fruit of *M. charantia* was suggested to modulate signal transduction pathways for inhibition of breast cancer cell growth [13]. Data from *in vitro* studies suggest that alpha- and beta-mormorcharin exert possible anti-herpes-virus effects [14], while momordin, a protein found in *M. charantia*, has anticancer activity in animal experiment [15]. More recently, MAP30, a 30-kDa protein isolated from seeds of* M. charantia*, has shown promising effects for treating tumors and HIV infection [16, 17]. In addition, cytotoxicity of RNases, ribosome inactivating protein, and triterpenoids isolated from the fruit of *M. charantia* on tumor cells has been demonstrated by numerous *in vitro* and *in vivo* studies [18–20]. 

Our preliminary assays indicated that extracts of *M. charantia* leaves obtained from eastern area of Taiwan were effective on inhibiting the growth of cancer cells. Hence the bioactivity of *M. charantia* is determined by extraction process and cultivars. To elucidate the antitumor activity of *M. charantia* with introgressed characteristics between cultivars and wild populations in the eastern Taiwan, we comparatively examined the effect of *M. charantia* methanol extract (MCME) by different human cancer cell lines in this study.

## 2. Materials and Methods

### 2.1. Preparation of *M. charantia* Methanol Extracts

*M. charantia* cultivated in the Hualien agriculture research and extension station (HARES, Hualien, Taiwan) with introgressed characteristics between cultivars and wild populations was authenticated before being used for this study. The plant material collected was identified by HARES, where a voucher specimen (no. 2381) has been deposited. The air-dried leaves of *M. charantia* were soaked in methanol at room temperature for 2 months, filtered and centrifuged at 500 ×g for 10 min. The supernatant was evaporated under reduced pressure to afford a dark brown residue, which was lyophilized at −80°C. The dried extract of *M. charantia* was stored at −20°C until required for treatments and dissolved in dimethyl sulfoxide with a stock concentration of 200 mg/mL before dilution with media.

### 2.2. Chemicals, Drugs, and Antibodies

Bovine serum albumin, 3-(4,5-dimethylthiazol-z-yl)-2,5-di-phenyl tetrazolium bromide (MTT), agarose, dimethylsulfoxide (DMSO), DMEM medium, penicillin, streptomycin, L-glutamine, sodium bicarbonate, trypsin/EDTA, propidium iodide (PI), DAPI, RNase A, Triton X-100, HEPES, NaOH, NaCl, EDTA, NP-40, Tris, sucrose, SDS, sodium deoxycholate, glycerol, Tween-20 were purchased from Sigma Chemical Company Inc. (St Louis, MO, USA). Anti-ICAD (113416), anti-caspase 3 (123678), anti-PARP (100573), anti-Bax (109683), anti-Bcl-2 (100064), and anti-*β*-actin (100315) were purchased from GeneTex Inc. (ICON-GeneTex, Hsinchu, Taiwan).

### 2.3. Cell Culture

To evaluate the antitumor properties of *M. charantia*, four human cancer cell lines, human nasopharyngeal carcinoma cells (Hone-1), gastric adenocarcinoma cells (AGS), colonrectal carcinoma cells (HCT-116), and lung adenocarcinoma cell (CL1-0), were used in this study. Cells were grown in DMEM, F-12 K, Mccoy's 5a and RPMI for Hone-1, AGS, HCT-116, and CL1-0 cells, respectively. All cultured media were supplemented with 10% FBS, 100 U/mL penicillin-100 *μ*g/mL streptomycin, and 0.1 M sodium bicarbonate. Cells were maintained in a humidified incubator at 37°C under 5% CO_2_.

### 2.4. Cell Viability Assay

Cytotoxicity of MCME on human cancer cells was assessed by MTT which measures the metabolic activity of viable cells as described [21]. Briefly, cells were plated out at a density of 5 × 10^3^ cells/well in 96-well microtiter plates. Following overnight cell adherence, fresh medium along with the corresponding concentrations of MCME were added to the culture. Cultural media were replaced by drug-free medium and MTT solution at a final concentration of 0.5 mg/mL after treatments, and incubation was prolonged for 4 h at 37°C. After carefully removing the supernatants, the MTT-formazan crystals formed by metabolically viable cells were dissolved in DMSO and absorbance was determined at 570 nm in a multiwell plate ELISA reader (Bio-tek Instruments, Winooski, VT, USA). The MCME concentration that caused approximate 25% and 50% growth inhibition was calculated, respectively, from extrapolating in the trend line by using the optical density OD value of control and the treated cells.

### 2.5. Propidium Iodide Staining of DNA Content

All cancer cells were seeded with an appropriate density in petri dishes and allowed to grow for 24 to 48 h at 37°C, in a condition of 5% CO_2_/95% air. Cells were harvested after treatments and fixed overnight with 70% ethanol at −20°C. Cells containing apoptotic bodies were counted under fluorescence microscopic observation (IX71, Olympus Co.).

For cell cycle analysis, cells were washed twice with PBS, and resuspended in 100 *μ*L of PI solution for 30 min at room temperature in the dark. Distribution of cells with different DNA contents was analyzed by a FACSCalibur flow cytometer and CellQuest software (BD Biosciences, San Jose, CA, USA) at an excitation wavelength of 530 nm. Fluorescence emission was measured using a 620 nm band pass filter.

### 2.6. DNA Fragmentation Assay

DNA fragments from cancer cells treated with MCME for 6, 12, 18, and 24 h were analyzed by agarose gel electrophoresis. Apoptotic DNA was isolated using DNA lysis buffer through the processes described previously [22]. Isolated DNA, mainly derived from the apoptotic bodies occurred in cells, was subjected to 2.0% agarose electrophoresis at 50 V for 3 h. DNA fragments, consisting of multimers of 160–200 base pairs, were visualized under ultraviolet light after staining with ethidium bromide.

### 2.7. Nuclear Staining

After treatment with MCME, cancer cells were fixed with 4% paraformaldehyde by 0.1% Triton X-100 and stained with 2 *μ*g/mL of 4,6-diamidine-2-phenylindole (DAPI) for 30 min at room temperature. Cells were washed twice with PBS and morphologic changes of nuclei with apoptosis characteristic were determined and counted by fluorescence microscopy (IX71, Olympus Co.).

### 2.8. Western Blot

Cells were harvested after various treatments and lysed with lysis buffer, containing 1 mM EDTA, 150 mM NaCl, 100 *μ*g/mL PMSF, 50 mM Tris-HCl (pH = 7.5), protease and phosphatase inhibitor cocktails (Sigma Co., MO, USA), and incubated on ice for 5 min. After centrifugation for 15 min at 4°C, the supernatant was transferred to fresh tube and stored at −20°C. Protein concentrations were determined using the Bradford protein assay reagent (Bio-Rad, CA, USA). For western blot analysis, equal amount of total protein was mixed with SDS sample buffer, incubated at 100°C for 5 min and separated by SDS-polyacrylamide gel electrophoresis. After electrophoresis, protein was blotted on a PVDF membrane (Millipore Co., Bedford, MA, USA) and blocked for 1 h in blocking solution at room temperature. Each membrane was incubated with appropriate primary antibodies at 4°C overnight and washed with PBST. The blots were incubated with the HRP-conjugated secondary antibodies at room temperature for 1 h, washed three times with PBST, and then followed by visualization with Immobilon western (Millipore Co., Bedford, MA, USA). 

### 2.9. Statistical Analysis

Quantified expression of proteins in all experiments was conducted using a densitometer (Personal Densitometer SI, Molecular Dynamics, Sunnyvale, CA, USA). All data were calculated as mean ± SD. Statistical analysis of group differences was conducted using the one way ANOVA and the Tukey's post hoc test for multiple comparisons. A value of *P* < 0.05 was considered statistically significant.

## 3. Results

### 3.1. Inhibition of Human Cancer Cell Growth by MCME

**Figure 1:**
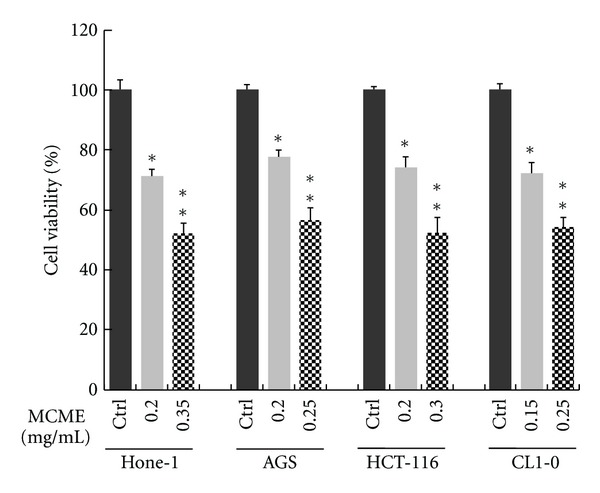
Inhibitory effect of MCME on the viability of cancer cells. Hone-1, AGS, HCT-116, and CL1-0 cells were used to evaluate the anticancer activity of MCME. Cell viability was determined by dose-response curves obtained by the MTT assay. To comparatively evaluate the susceptibility of cells to MCME, data of each cell line were shown only at concentrations ranging from 0.15 to 0.35 mg/mL for the approximate viability of 75% and 50% at 24 h for each. All experiments were performed in triplicate, and results are expressed as mean ± SD at a sample number of 8 for each experiment. (*) and (**) indicate significant *P* values <0.05 and <0.01, respectively, as compared to control (Ctrl).

The effect of MCME on cell survival in four human cancer cell lines was evaluated for 24 h by an MTT assay. As shown in Figure 1, Hone-1, AGS, HCT-116, and CL1-0 were exposed to 0.15 ~ 0.35 mg/mL MCME for 24 h. MCME exhibited cytotoxic activity in all cancer cell lines tested, displaying a minor difference of IC_50_. The inhibitory effects were similar in Hone-1 cells (estimated IC_50_, 0.35 mg/mL), AGS cells (estimated IC_50_, 0.3 mg/mL), HCT-116 cells (estimated IC_50_, 0.3 mg/mL), and CL1-0 cells (estimated IC_50_, 0.25 mg/mL). Approximately 50% of cancer cells survived after exposure for 24 h in each cell line, as follows: 52.0 ± 3.5% for Hone-1 cells, 56.5 ± 4.2% for AGS cells, 52.3 ± 5.1% for HCT-116 cells, and 54.2 ± 3.2% for CL1-0 cells. Therefore, the susceptibility of these cancer cells to MCME was considered to be similar. 

### 3.2. Effects of MCME on Apoptosis Induction

**Figure 2:**
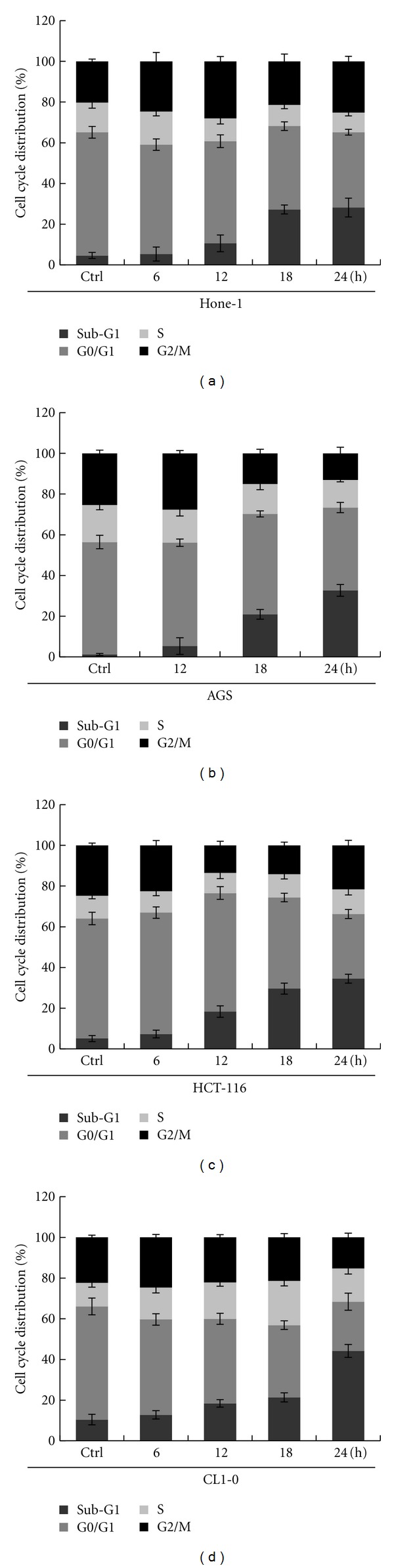
Cell cycle distribution of MCME-treated cancer cells. Cells were treated with MCME for 6, 12, 18, and 24 hours at a concentration of 0.35, 0.25, 0.3, and 0.25 mg/mL for Hone-1, AGS, HCT-116, and CL1-0 cells, respectively. MCME-treated cells were analyzed by flow cytometry using PI staining to determine the population at sub-G1 phase. All experiments were performed in triplicate, and results were expressed as mean ± SD. Ctrl indicates the control group of cells.

The cytotoxic effects of MCME on these cancer cells were investigated by examining the cell cycle distribution using PI staining. The quantitative data indicated that exposure to MCME induced an increased amount of cells at sub-G1 phase, whereas a reduced amount of cells in S phase to total cells, in all cancer cell lines. After treatment with MCME for 24 h, the sub-G1 population increased from 4.6% to 28.2% in Hone-1 cells, from 2.1% to 44.5% in AGS cells, from 5.1% to 34.5% in HCT-116 cells, and from 10.5% to 44.2% in CL1-0 cells (Figure 2). 

**Figure 3:**
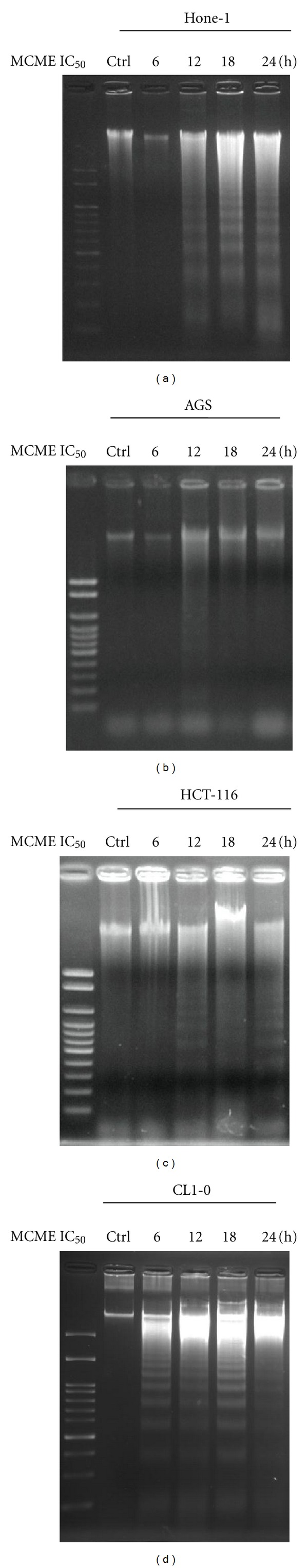
Induction of DNA fragmentation by MCME in cancer cells. Cells were treated at a number of 1 × 10^6^ with MCME for 6, 12, 18, and 24 hours at a concentration of 0.35, 0.25, 0.3, and 0.25 mg/mL for Hone-1, AGS, HCT-116, and CL1-0 cells, respectively. The DNA fragmentation of cells was detected on a 2% agarose gel electrophoresis. DNA fragmentation was visualized by Ethidium Bromide (EtBr) under UV light. Ctrl indicates the control group of cells.

The increased sub-G1 population indicated a less amount of MCME-treated cancer cells entering cell cycle after MCME treatment. However, cell population at sub-G1 phase was much less than that determined by cell viability assay. To further evaluate MCME induced cell death in these cancer cells, DNA ladder pattern of internucleosomal DNA fragments was analyzed as shown in Figure 3. DNA fragmentation was observed in MCME-treated Hone-1, AGS, and HCT-116 cells from 12 h after treatment, whereas a significant ladder pattern of DNA fragmentation was found at 6 h in CL1-0 cells. These results indicated that MCME resulted in DNA fragmentation in cancer cells and the susceptibility of CL1-0 cells to MCME induced cell death was higher than that of Hone-1, AGS, and HCT-116 cells. 

**Figure 4:**
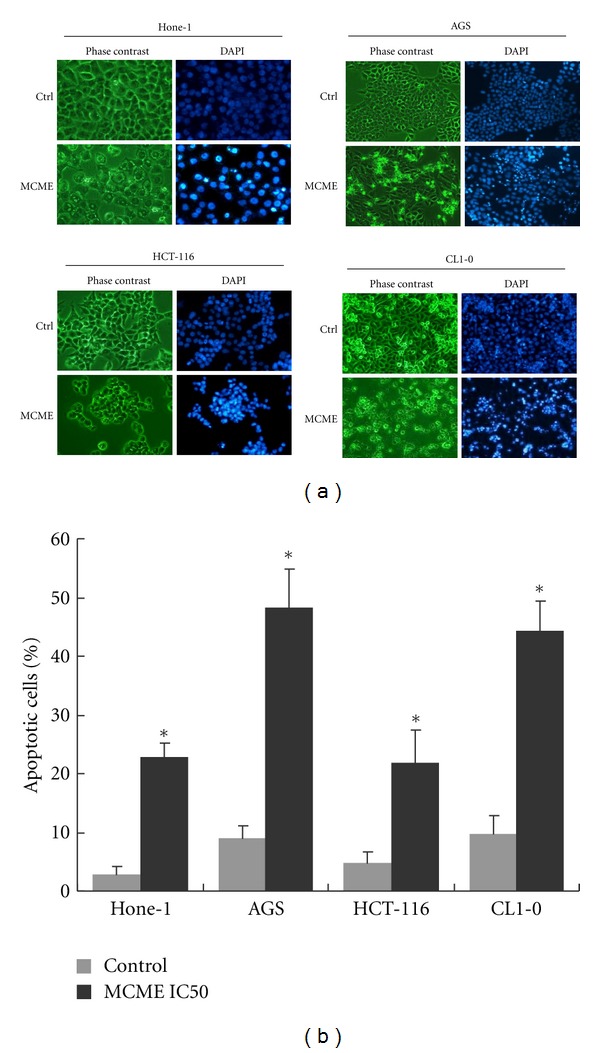
Analysis of the apoptosis in MCME-treated cancer cells. (a) Morphological changes of Hone-1, AGS, HCT-116, and CL1-0 cells treated with MCME at 0.35, 0.25, 0.3, and 0.25 mg/mL for 24 h. (b) Assessment of apoptotic cells in MCME-treated cancer cells. Cells with apoptotic characteristic DAPI-staining nuclei were counted by at least 1000 cells using fluorescence microscopy. Data were represented by mean ± SD from three independent experiments. Asterisk (*) indicates a significant *P* value < 0.05, as compared with controls.

In addition, DNA damage was observed in all cell lines, as indicated by morphological changes in nuclei. It indicated that MCME caused marked apoptotic changes characterized by nuclear shrinkage and chromatin condensation (Figure 4(a)), which were quantified as shown in Figure 4(b). The increased percentage of apoptotic cells found after treatment with MCME for 24 h, coincided with that evaluated by sub-G1 populations (Figure 2).

### 3.3. MCME-Induced Apoptosis through Caspase-Dependent Pathway

**Figure 5:**
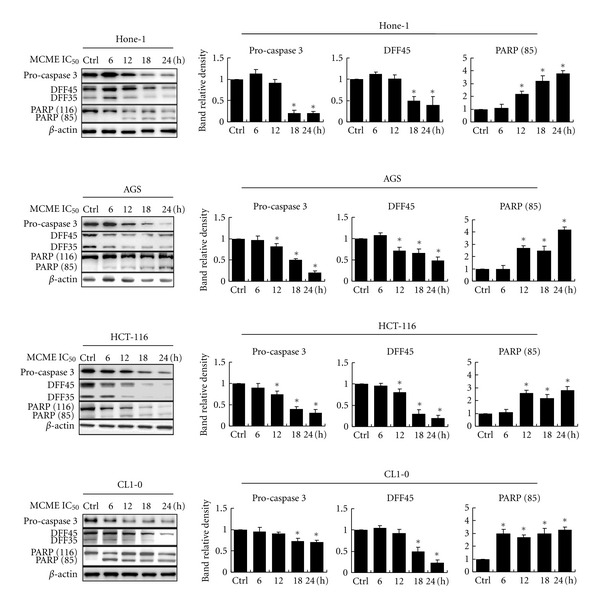
Expression of apoptogenic proteins in MCME-treated cancer cells. Hone-1, AGS, HCT-116, and CL1-0 cells were treated for 6, 12, 18, and 24 hours with MCME at a concentration of 0.35, 0.25, 0.3, and 0.25 mg/mL, respectively. Equal amounts of lysates from these cancer cells treated with MCME were immunoblotted with anti-pro-caspase 3, anti-DFF, and anti-PARP antibodies. Blots were reproved with an antibody for *β*-actin to control for protein loading and transfer. Gels shown are representative of those obtained from three independent experiments. The protein expression levels were quantified and normalized to *β*-actin and expressed as the fold-change to the respective control. Asterisk (*) indicates a significant *P* value < 0.05 as compared to controls.

To explore the mechanism of MCME-induced apoptosis, activation of caspase and expression of proapoptogenic proteins were analyzed by western blotting. Caspase-3 is one of the hallmarks of apoptosis and is responsible for inducing apoptosis by cleaving a variety of substrates such as DFF-45 (DNA fragmentation factor 45, also known as ICAD) and poly-(ADP-ribose) polymerase (PARP) [23, 24]. DFF-45 is the inhibitor of DFF-40 (also known as CAD), which is responsible for double-stranded DNA cleavage in apoptosis. The functional loss of DFF-45 allows DFF-40 to dimerize into the catalytic form, inducing DNA fragmentation [25]. To identify the mechanism involved in MCME-induced apoptosis, we investigated the activation of caspase-3 and DFF-45 in cancer cells treated with MCME for 24 h. MCME significantly decreased the activation of procaspase-3 and the cleavage of DFF-45 (endogenous substrate of caspase-3) in a time-dependent manner (*P < 0.05) (Figure 5). These findings suggest that the activation of caspases involved in the apoptotic pathway is one of the major mechanisms by which MCME affects human cancer cell lines.

### 3.4. Effect of MCME on the Cleavage of PARP

PARP is a key signaling nuclear protein involved in triggering DNA repair [26]. This enzyme can catalyze poly (ADP-ribose) ligation to an acceptor protein, including itself. During apoptosis, PARP is cleaved by the activation of caspase-3, resulting in DNA damage and apoptosis [27]. As PARP is a downstream substrate of caspase-3, cleavage of PARP is an indicator of apoptosis. Treatment with MCME in all cancer cell lines resulted in the cleavage of PARP to yield an 85-kDa cleaved fragment (Figure 5). After treatment with MCME for 24 h, PARP (85) showed a 3 ~ 5-fold increase of activity at the approximate IC_50_ concentrations in all cancer cells (*P < 0.05).

### 3.5. MCME-Mediated Expression of Bax and Bcl-2

**Figure 6:**
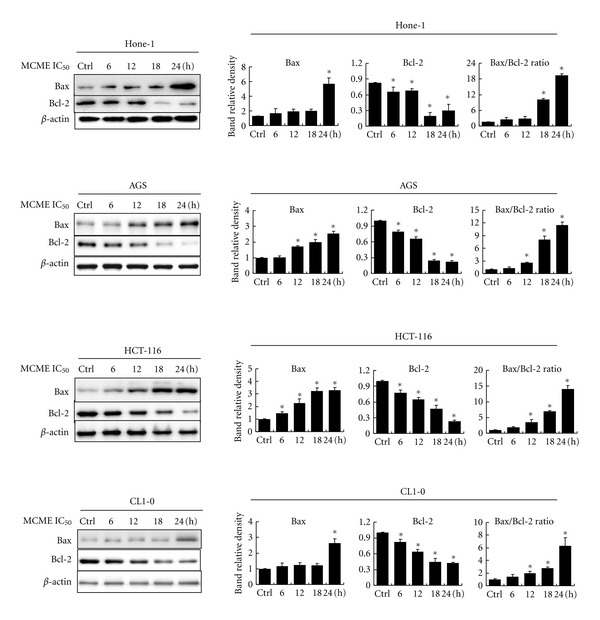
Expression of Bcl-2 family proteins in MCME-treated cancer cells. Hone-1, AGS, HCT-116, and CL1-0 cells were treated for 6, 12, 18, and 24 hours with MCME at a concentration of 0.35, 0.25, 0.3, and 0.25 mg/mL, respectively. Equal amounts of lysates from these cancer cells treated with MCME were immunoblotted with anti-Bax and anti-Bcl-2 antibodies. Blots were reproved with an antibody for *β*-actin to control for protein loading and transfer. Gels shown are representative of those obtained from three independent experiments. The protein expression levels were quantified and normalized to *β*-actin and expressed as the fold-change to the respective control. Asterisk (*) indicates a significant *P* value < 0.05 as compared to controls.

Bcl-2 family proteins have a central role in controlling the mitochondrial pathway. The Bcl-2 family significantly regulates apoptosis either as an activator or as an inhibitor. It has been suggested that the Bax/Bcl-2 ratio was a key factor in regulation of the apoptotic process [28, 29]. As shown in Figure 6, expression of proapoptotic Bax was significantly increased, whereas the antiapoptotic Bcl-2 protein was decreased after treatment with MCME in a time-dependent manner. MCME-induced the increased expression of Bax and decreased expression of Bcl-2, leading to a consequential increase of Bax/Bcl-2 ratio. Bax/Bcl-2 ratio was significantly increased by treating with MCME for 18 h and was increased with a range from 6.3 to 19.4 at 24 h on different cancer cells. The initiation of apoptosis through mitochondrial membrane permeability by the increased Bax/Bcl-2 ratio indicates that apoptosis induced by MCME is mitochondria related.

## 4. Discussion

Plant-derived herbal medicines have been used for a long time in several Asian countries. Anticancer activity is one among all the effects been reported by studies *in vitro* using natural herb extracts [30, 31]. *M. charantia*, also called bitter melon, is a popular vegetable in Asia. The ability of this plant to decrease blood sugar was recognized more than 600 years ago [32]. Recent research confirms the presence of proteins and metabolites in *M. charantia* that exhibit hypoglycemic effect and act as antitumor and antiviral agents [9, 33, 34]. For instance, Kuguacin J and momorcharins were effective against human prostate cancer [35, 36]. These findings suggest that* M. charantia* has great potential as a health food or a source for new drug development. In the current study, we extended the antitumor potential of *M. charantia* to other different cancers. Same as studies reported previously, MCME exhibited cytotoxicity on Hone-1, AGS, HCT-116, and CL1-0 cells, which was attributed to its action on the induction of cell death. 

Apoptosis is a programmed cell death that can occur by a variety of internal or external stimuli, and these signals are controlled by two distinct pathways. One is an extrinsic pathway (death receptor pathway), and the other is an intrinsic pathway in which mitochondria are involved [37, 38]. Caspases play a key role in apoptosis [39, 40]. A regulatory cascade eventually leads to the activation of effector caspases, such as caspase-3. These caspases are responsible for the cleavage of cellular proteins, such as cytoskeletal components, leading to the typical morphological changes observed in cells undergoing apoptosis [41]. Our results indicated that MCME increased caspase-3 activity in Hone-1, AGS, HCT-116, and CL1-0 cells in a time-dependent manner. Programmed cell death involves the activation of caspases and the subsequent cleavage of several substrates such as actin, PARP, gelsolin, DFF-45, lamins, and fodrin [42]. PARP is involved in repairing DNA damage by catalyzing the synthesis of poly (ADP-ribose), binding to DNA strand breaks, and modifying nuclear proteins [23, 24, 43]. The ability of PARP to repair DNA damage is prevented by cleavage of PARP by caspase-3 [44]. The fragmentation of DNA into nucleosomal units as seen in DNA fragmentation assays is caused by an enzyme known as DFF-40, or caspase-activated DNase. Normally, DFF-40 exists as an inactive complex with its inhibitor, DFF-45. During apoptosis, DFF-45 is cleaved by caspases, such as caspase-3, to release DFF-40 and followed by rapid fragmentation of the nuclear DNA [45, 46]. 

In this study, we found that MCME exhibited a significant effect on the morphological and biochemical features of the cancer cells tested, indicating that MCME induces apoptosis in both dose- and time-dependent manner. We observed that treatment of cells with up to 0.15 ~ 0.2 mg/mL MCME reduced one fourth of cell viability, whereas about a half of cell viability was found at 0.25 ~ 0.35 mg/mL. MCME significantly inhibited cell viability and induced chromatin condensation in Hone-1, AGS, HCT-116, and CL1-0 cells. In addition, MCME induced the cleavage of caspase-3 and DFF-45, and the activation of PARP, leading to DNA fragmentation. Among all the tested cancer cells, CL1-0 cells were more susceptible to MCME with a lower IC_50_ and the apace increased cleavage of PARP and DNA fragmentation at 6 h after treatment. Cells containing apoptotic bodies confirmed that MCME-induced cell death and sub-G1 populations in CL1-0 and AGS cells were mainly mediated through caspase-dependent apoptotic pathway. However, the lower population of cells with apoptotic bodies and sub-G1 phase distribution imply some other pathways may be involved in MCME-reduced viability in Hone-1 and HCT-116 cells. 

Both biochemical and genetic evidence indicates that Bcl-2 family members, including proapoptotic proteins such as Bax, Bak and antiapoptotic proteins such as Bcl-2, Bcl-xl, can regulate cell death induced by caspases [47, 48]. In recent years, the importance of Bcl-2 family members in mitochondria-associated apoptotic network is noticed [49]. Bcl-2, a caspase substrate, can function as an antioxidant to inhibit apoptosis in a wide variety of cell types, which play a vital role in regulating the mitochondria-dependent pathway [50]. The Bax/Bcl-2 ratio can be recognized as a key factor for apoptotic process by regulating cytochrome *c* from mitochondria to cytosol [51, 52]. Extract of *M. charantia* was reported to inhibit growth of several cancer cells by augmenting Bax/Bcl-2, Bad/Bcl-2, or Bak/Bcl-2 [36, 53]. Our results showed that Bcl-2 was significantly decreased and Bax was increased in MCME-treated cancer cells. The increased Bax/Bcl-2 ratio indicates that mitochondria-mediated apoptosis is involved in MCME-induced cell death in these treated cell lines [49]. The activation of caspase-3 and enhanced production of cleaved PARP, subsequently leading to DNA fragmentation and apoptosis, in MCME-treated cancer cells coincided with that treated by Kuguacin J, a triterpenoid, and Rutin, a flavonoid, from leaf of *M. charantia*, in leukemia, prostate, and ovarian cancer cells [36, 54, 55]. Therefore, both Kuguacin J and Rutin may be the active components exhibiting anti-cancer activity in MCME. Interestingly, our results on Hone-1 nasopharyngeal carcinoma cells did not meet the unchanged Bcl-2 expression in *M. charantia *lectin-induced apoptosis in another nasopharyngeal carcinoma CNE-1 cells [19]. It indicates that MCME mediated different processes in cancer cells toward apoptosis.

Owing to the edible properties of *M. charantia*, two cancer cell lines of digestive track, AGS and HCT-116 cells, were used to comparatively investigate the anticancer effect of *M. charantia* on two other cancer cell lines, Hone-1 and CL1-0 cells, in this study. In spite to the difference of MCME-induced cell viability, all these cells represent a susceptibility to MCME-induced cell death through caspase- and mitochondria-dependent apoptotic pathways. Since people consume fruit and/or leaves of *M. charantia* as food in Asia, MCME used in this study represents a promising candidate agent to develop for cancer prevention in the future.

## 5. Conclusion

In conclusion, we demonstrated that caspase- and mitochondria-dependent pathways are involved in MCME-induced apoptosis in Hone-1, AGS, HCT-116, and CL1-0 cancer cells. Since a lot of processes may be initiated, cell death other than apoptosis is probably involved in death stimuli in these cancer cells by MCME. Edible materials possess anti-cancer properties, may be particularly useful for synergistic remedy with conventional drugs to circumvent drug resistance in cancer therapy. MCME that can initiate anti-cancer actions through caspase- and mitochondria-dependent pathways exhibits the potential for complementary therapy on cancers.
